# MEANINGFUL ACTIVITY IN ASSISTED LIVING RESIDENTS WITH DEMENTIA: RESULTS FROM THE MAC-4-BSD STUDY

**DOI:** 10.1093/geroni/igae098.4239

**Published:** 2024-12-31

**Authors:** Sarah Holmes, Elizabeth Galik, Barbara Resnick, Susan Scherr, Erin O’Brien, Sorah Levy, Merve Gurlu, Shijun Zhu

**Affiliations:** University of Maryland School of Nursing, Baltimore, Maryland, United States; University of Maryland School of Nursing, Baltimore, Maryland, United States; University of Maryland School of Nursing, Baltimore, Maryland, United States; Stevenson University, Owings Mills, Maryland, United States; Post-Acute and Long-Term Care Medical Association, Columbia, Maryland, United States; University of Maryland School of Nursing, Baltimore, Maryland, United States; University of Maryland School of Nursing, Baltimore, Maryland, United States; University of Maryland School of Nursing, Baltimore, Maryland, United States

## Abstract

Engagement in meaningful activity is beneficial for assisted living (AL) residents with dementia and may help to reduce behavioral symptoms and improve quality of life. The purpose of this study was to test the feasibility and preliminary efficacy of a theory-based approach, Meaningful Activity for Managing Behavioral Symptoms of Distress (MAC-4-BSD), for decreasing behavioral symptoms and increasing engagement in meaningful activity. It was hypothesized that residents exposed to MAC-4-BSD will demonstrate reduced behavioral symptoms, increased meaningful activity engagement and quality of life at 4 months compared to those exposed to education only. Using a cluster randomized controlled trial with repeated measures design, this study included 71 residents with dementia from 5 communities. Residents were evaluated at baseline and 4 months. Descriptive statistics were used to describe the sample and evaluate feasibility. Linear mixed models were used to determine effectiveness. Most participants were female (n=52, 73%), and White (n=62, 87%) with a mean age of 85 years (SD=8.2). The MAC-4-BSD intervention was feasible to implement with regard to delivery, receipt, and enactment. Preliminary effectiveness was demonstrated based on increased engagement in meaningful activity (b=7.69, p=.005), improved quality of life (b=3.74, p=.03), and decreased staff distress related to residents’ behavioral symptoms (b=-1.83, p=.037) among those exposed to MAC-4-BSD versus the control group. There was no change in the severity or number of behavioral symptoms. These findings demonstrate that engagement in meaningful activity is a promising approach that can be used to help improve the quality of life for AL residents with dementia.

